# Supplementary material for: Lipoprotein Profile in Populations from Regions of the Russian Federation: ESSE-RF Study
Source: Int J Environ Res Public Health. 2022 Jan 14;19(2):931. doi: 10.3390/ijerph19020931 (PMC8775951; doi:10.3390/ijerph19020931)
Supplement: Supplementary file 1 [file ijerph-19-00931-s001.zip › ijerph-1503226-SI.pdf]

Supplement-Table S1. Sex- and age specific values of lipoprotein profile of lipoprotein profile parameters among populations from regions of Russian Federation: ESSE-RF study

Age, years	Total cohort	Men	Women	p
		Total cholesterol (M±SD), mmol/L		
25-34	4.77 ± 1.00 [b,c,d]<br>n = 4413	4.84 ± 1.04 [b,c,d]<br>n = 2076	4.72 ± 0.96 [b,c,d]<br>n = 2337	0.000
35-44	5.23 ± 1.06 [a,c,d]<br>n = 4214	5.38 ± 1.14 [a,c,d]<br>n = 1693	5.13 ± 0.99 [a,c,d]<br>n = 2521	0.000
45-54	5.62 ± 1.14 [a,b,d]<br>n = 5919	5.55 ± 1.17 [a,b,d]<br>n = 2056	5.66 ± 1.13 [a,b,d]<br>n = 3863	0.000
55-64	5.78 ± 1.20 [a,b,c]<br>n = 6619	5.46 ± 1.15 [a,b,c]<br>n = 2113	5.93 ± 1.20 [a,b,c]<br>n = 4506	0.000
All	5.42 ± 1.18<br>n = 21165	5.30 ± 1.16<br>n = 7938	5.48 ± 1.19<br>n = 13227	0.000
		LDL-cholesterol (M±SD), mmol/L		
25-34	2.87 ± 0.87 [b,c,d]<br>n = 4413	3.00 ± 0.88 [b,c,d]<br>n = 2076	2.76 ± 0.85 [b,c,d]<br>n = 2337	0.000
35-44	3.24 ± 0.93 [a,c,d]<br>n = 4214	3.41 ± 0.98 [a,c,d]<br>n = 1693	3.13 ± 0.89 [a,c,d]<br>n = 2521	0.000
45-54	3.56 ± 1.00 [a,b,d]<br>n = 5919	3.55 ± 1.01 [a,b]<br>n = 2056	3.56 ± 0.99 [a,b,d]<br>n = 3863	0.738
55-64	3.70 ± 1.04 [a,b,c]<br>n = 6619	3.50 ± 1.00 [a,b]<br>n = 2113	3.79 ± 1.04 [a,b,c]<br>n = 4506	0.000
All	3.40 ± 1.02<br>n = 21166	3.36 ± 0.99<br>n = 7939	3.42 ± 1.04<br>n = 13227	0.003
		Triglycerides (Me [Q1; Q3]), mmol/L		
25-34	0.93 [0.68; 1.34] [b,c,d]<br>n = 4413	1.06 [0.75; 1.56] [b,c,d]<br>n = 2076	0.83 [0.63; 1.16] [b,c,d]<br>n = 2337	0.000
35-44	1.09 [0.78; 1.6] [a,c,d]<br>n = 4214	1.27 [0.90; 1.98] [a,c,d]<br>n = 1693	0.99 [0.73; 1.4] [a,c,d]<br>n = 2521	0.000
45-54	1.27 [0.91; 1.84] [a,b,d]<br>n = 5919	1.37 [0.97; 2.04] [a,b]<br>n = 2056	1.21 [0.88; 1.74] [a,b,d]<br>n = 3863	0.000
55-64	1.39 [1.02; 1.96] [a,b,c]<br>n = 6618	1.35 [0.98; 1.95] [a,b]<br>n = 2113	1.40 [1.04; 1.96] [a,b,c]<br>n = 4505	0.013
All	1.20 [0.85; 1.74]<br>n = 21165	1.26 [0.89; 1.88]<br>n = 7939	1.16 [0.82; 1.67]<br>n = 13226	0.000
		HDL-cholesterol (M±SD), mmol/L		
25-34	1.40 ± 0.35<br>n = 4413	1.29 ± 0.32<br>n = 2076	1.49 ± 0.34 [d]<br>n = 2337	0.000
35-44	1.42 ± 0.36[d]<br>n = 4214	1.31 ± 0.34<br>n = 1693	1.49 ± 0.36 [d]<br>n = 2521	0.000
45-54	1.41 ± 0.36<br>n = 5919	1.28 ± 0.34<br>n = 2056	1.48 ± 0.35 [d]<br>n = 3863	0.000
55-64	1.39 ± 0.34 [b]<br>n = 6619	1.30 ± 0.33<br>n = 2113	1.44 ± 0.34 [a,b,c]<br>n = 4506	0.000
All	1.40 ± 0.35<br>n = 21166	1.29 ± 0.33<br>n = 7939	1.47 ± 0.35<br>n = 13227	0.000
		Lp(a) (Me [Q1; Q3]), mg/dL		

25-34	8.00 [3.70; 20.10] [b,c,d] n = 2093	7.60 [3.50; 19.33] [b,c,d] n = 980	8.50 [3.80; 21.00] [b,c,d] n = 1113	0.084
35-44	10.00 [4.20; 23.90] [a,c,d] n = 2137	10.45 [4.20; 25.62] [a] n = 838	9.90 [4.30; 23.35] [a,c,d] n = 1299	0.949
45-54	12.20 [5.18; 27.90] [a,b,d] n = 2972	10.70 [4.70; 22.90] [a] n = 959	12.90 [5.30; 29.50] [a,b,d] n = 2013	0.000
55-64	13.30 [6.00; 31.02] [a,b,c] n = 3228	11.40 [4.80; 26.40] [a] n = 986	13.90 [6.50; 33.70] [a,b,c] n = 2242	0.000
All	11.10 [4.80; 26.80] n = 10430	9.80 [4.20; 22.9] n = 3763	11.70 [5.15; 28.10] n = 6667	0.000
		apo AI (M±SD), g/L		
25-34	1.58 ± 0.4 n = 2371	1.50 ± 0.37 n = 1103	1.65 ± 0.42 n = 1268	0.000
35-44	1.60 ± 0.41 n = 2417	1.53 ± 0.39 n = 948	1.64 ± 0.41 n = 1469	0.000
45-54	1.60 ± 0.43 n = 3381	1.53 ± 0.53 n = 1098	1.63 ± 0.38 n = 2283	0.000
55-64	1.58 ± 0.36 n = 3637	1.50 ± 0.35 n = 1107	1.61 ± 0.36 n = 2530	0.000
All	1.59 ± 0.40 n = 11807	1.52 ± 0.43 n = 4257	1.63 ± 0.38 n = 7550	0.000
		apo B (M±SD), g/L		
25-34	0.77 ± 0.21 [b,c,d] n = 2378	0.80 ± 0.22 [b,c,d] n = 1107	0.74 ± 0.20 [b,c,d] n = 1271	0.000
35-44	0.87 ± 0.24 [a,c,d] n = 2424	0.92 ± 0.24 [a,c,d] n = 951	0.83 ± 0.22 [a,c,d] n = 1473	0.000
45-54	0.96 ± 0.25 [a,b,d] n = 3388	0.97 ± 0.25 [a,b] n = 1099	0.95 ± 0.24 [a,b,d] n = 2289	0.262
55-64	1.01 ± 0.26 [a,b,c] n = 3660	0.96 ± 0.25 [a,b] n = 1117	1.03 ± 0.27 [a,b,c] n = 2543	0.000
All	0.92 ± 0.26 n = 11851	0.91 ± 0.25 n = 4275	0.92 ± 0.26 n = 7576	0.351
		apo B/apo AI (Me [Q1; Q3])		
25-34	0.48 [0.37; 0.62] [b,c,d] n = 2371	0.52 [0.41; 0.67] [b,c,d] n = 1103	0.44 [0.35; 0.56] [b,c,d] n = 1268	0.000
35-44	0.54 [0.42; 0.70] [a,c,d] n = 2417	0.61 [0.47; 0.78] [a,c,d] n = 948	0.51 [0.4; 0.64] [a,c,d] n = 1469	0.000
45-54	0.61 [0.47; 0.77] [a,b,d] n = 3380	0.65 [0.49; 0.83] [a,b] n = 1097	0.59 [0.45; 0.75] [a,b,d] n = 2283	0.000
55-64	0.65 [0.50; 0.82] [a,b,c] n = 3637	0.66 [0.49; 0.83] [a,b] n = 1107	0.65 [0.50; 0.82] [a,b,c] n = 2530	0.716
All	0.58 [0.44; 0.75] n = 11806	0.60 [0.46; 0.78] n = 4256	0.56 [0.43; 0.73] n = 7550	0.000

[a, b, c, d] – $p<0.05$, where the group aged 25-34 is indicated by [a], aged 34-44 – by [b], aged 45-54 – by [c], and aged 55-64 – by [d]; p – significance of differences between men and women
